# Estimating changes of forest carbon storage in China for 70 years (1949–2018)

**DOI:** 10.1038/s41598-023-44097-4

**Published:** 2023-10-06

**Authors:** WeiSheng Zeng, XinYun Chen, XueYun Yang

**Affiliations:** Academy of Inventory and Planning, National Forestry and Grassland Administration, Beijing, 100714 China

**Keywords:** Ecology, Mathematics and computing

## Abstract

In the realm of forest resource inventory and monitoring, stand-level biomass carbon models are especially crucial. In China, their importance is underscored as they form the bedrock for estimating national and international forest carbon storage. This study, based on the data from 52,700 permanent plots in the 9th National Forest Inventory (NFI) of China, was directed towards developing these models. After computing biomass and carbon storage per hectare using specific tree models for 34 species groups, we devised robust volume-derived biomass and carbon storage models for 20 forest types. The application of these models and historical data reveals notably a decline in China's forest carbon storage to 4.90Pg by the late 1970s due to aggressive forest exploitation. However, subsequent conservation and afforestation campaigns have affected a recovery, culminating in a storage of 8.69Pg by the 9th NFI. Over the past 40 years, China's forest carbon storage has surged by 3.79Pg, split between natural forests (2.25Pg) and planted forests (1.54Pg). In benchmarking against three pre-existing models, we discerned discernible biases, underscoring the need for larger modeling sample sizes. Overall, our models stand as a monumental stride in accurately gauging forest carbon storage fluctuations in China, both regionally and nationally.

## Introduction

Similar to forest volume, forest biomass and carbon storage are not only important indicators of forest resources monitoring at all levels, but they are also important parameters reflecting the function and productivity of forest ecosystem^1–3^. With the increasing focus on global climate change, there is growing attention to the research on forest carbon storage and carbon sequestration potential^4,5^. The estimate of forest biomass can be obtained either by developing individual tree biomass models^6,7^, or by establishing stand-level biomass models or biomass conversion factor models^2,6^. Forest carbon storage estimates can be derived by multiplying forest biomass with the average carbon factor of the forest^2^.

According to a review by Luo et al.^8^, Chinese scholars have published 5924 individual tree biomass models for nearly 200 tree species from 1978 to 2013. Since 2014, the State Forestry Administration has systematically developed tree biomass models and related parameters to carbon accounting for major tree species in China, and promulgated and implemented a series of ministerial standards^9–21^. However, both domestically and internationally, the published stand-level biomass models^7,22–41^ are significantly fewer than the tree-level biomass models^7,22,26,27,42–44^.

Among the published biomass models of various forest types in China, the most influential one is the volume-derived biomass model of 21 forest types proposed by Fang et al.^29,30^ based on the data of 418 sample plots, which are still cited in many studies^35,38^. Wang et al.^32^ established the hyperbolic models between forest biomass and volume of 16 forest types in China using the data of 1,266 plots from different forest types. Zhang et al.^41^ developed power function models between forest biomass and volume of 10 forest types in China by using the data of 1,828 sample plots from different forest types, and established 21 biomass models for seven regions and three forest types.

An analysis of the performance of these models reveals three main shortcomings: First, the number of samples used for modeling is generally low, with some models being based on an extremely small sample size. For example, among the 21 models developed by Fang et al.^30^, 18 models were based on fewer than 30 plots, and only three models for larch (*Larix* spp.), Chinese fir (*Cunninghamia lanceolata*) and Chinese pine (*Pinus tabulaeformis*) were based on more than 30 plots; Of the 16 models established by Wang et al.^32^ 10 models were based on less than 50 plots and three models even used only fewer than 10 plots; Among the 10 models developed by Zhang et al.^41^ there are also two models based on fewer than 50 plots. Second, the modeling method is not appropriate. They usually used ordinary least square (OLS) method, and did not consider the biomass and volume data having heteroscedastic features, thus ignoring the basic assumptions using OLS method to develop models. Third, the evaluation index is single. Only one evaluation index, R^2^ or R, was provided, and no other evaluation indices in terms of error were provided, so its applicability was doubtful.

Zhang et al.^40^ established 21 power function models between biomass and volume of individual trees according to three types in seven regions by using the data of 7533 sample trees for different tree species, and used them for biomass and carbon storage estimation of different forest types. Although the problem of insufficient sample size has been solved, scale inconsistency exists when tree-level models of different species are treated as stand-level models for carbon storage estimation of different forest types, thus scale conversion must be carried out by the appropriate approach.

As early as the beginning of this century, Fang et al.^30^ estimated the change of forest carbon storage in China from 1949 to 1998, based on the proposed volume-derived biomass models of 21 forest types and the results of national forest inventory (NFI) in the past 50 years, which decreased from 5.06Pg to 4.38Pg and then increased to 4.75Pg. Zhou et al.^37^ estimated the change of forest carbon storage in China from 1994 to 2013, ranging from 4.14Pg to 5.96Pg. Tang et al.^39^ estimated the forest carbon storage based on results of the 8th NFI (2009–2013) to reach 10.48Pg. In view of the wide difference in the estimation results of different scholars and different methods, Zhou et al.^38^ re-estimated the changes of forest carbon storage in China from 1973 to 2013 based on the wood density method, ranging from 3.0Pg to 5.9Pg. Zhang et al.^40^ estimated the changes of China's forest carbon storage from 1949 to 2018, using the data of previous NFIs and the developed biomass carbon storage models, which decreased from 4.38Pg to 3.69Pg and then increased to 7.97Pg. It can be seen that different scholars, to some extent, have different estimated results of forest carbon storage in the same period. The main influencing factors are the differences in both methods and data usage. Secondly, the model-based method had significant differences in modeling sample size and model uncertainty. Even though they were based on NFI data, the comparability of dynamic data could not be guaranteed due to incomparable factors that they did not know between the data of each period.

In this study, we have two objectives: (i) to develop volume-derived biomass and carbon storage models at the stand level for 20 forest types using an appropriate method, based on a large number of representative sample plots, and compare them with some published models; (ii) to estimate the forest carbon storages in different periods in China during the past 70 years and the carbon storages of planted forest in the last 40 years, using both the models developed in this study and the data of NFIs from the 1st to the 9th and the data of area and volume for different forest types in 1949 and 1950–1962, based on full consideration of various incomparable factors.

## Developing models of forest biomass and carbon storage

### Data description

The data employed for this study were sourced from the permanent sample plots recorded in the 9th National Forest Inventory (NFI). These plots were categorized into 20 distinct forest types based on their area and volume. These types encompass fir (*Abies*), spruce (*Picea*), larch (*Larix*), Chinese fir (*Cunninghamia lanceolata*), cypress (*Cupressus*), Masson pine (*Pinus massoniana*), Chinese pine (*Pinus tabulaeformis*), Yunnan pine (*Pinus yunnanensis*), other coniferous, oak (*Quercus*), birch (*Betula*), poplar (*Populus*), Robinia (*Robinia pseudoacacia*), eucalypt (*Eucalyptus*), rubber-woods, other hard-broadleaved species, other soft-broadleaved species, coniferous mixed forests, conifer-broadleaved mixed forests and broad-leaved mixed forests.

Across the nation, there are a total of 52,700 effective plots documented, all of which boast a forest volume greater than 0. Each of these plots underwent an assessment wherein the forest volume, biomass (including both above- and below-ground biomass but excluding the biomass of understory shrub and herbaceous layers), and carbon storage per hectare were meticulously calculated. This calculation utilized the one-variable tree volume and biomass models and incorporated the carbon factors of primary tree species^9–21^.

To ensure an effective modeling and validation process, the plots were systematically divided: two-thirds of the plots were designated for modeling, and the remaining one-third was allocated for validation purposes. Table 1 offers a comprehensive overview, detailing the basic data pertaining to the modeling samples and validation samples across the 20 forest types.

**Table 1:** The basic data pertaining to the modeling samples and validation samples across the 20 forest types.

Forest types	Number of plots	Modeling samples			Validation samples		
		Number of plots	Max volume (m^3^/ha)	Max biomass (t/ha)	Number of plots	Max volume (m^3^/ha)	Max biomass (t/ha)
Coniferous							
Fir	534	355	1439	764	179	1364	792
Spruce	1353	900	941	564	453	800	487
Larch	2495	1665	485	381	830	522	379
Chinese fir	3152	2100	456	257	1052	454	262
Cypress	1328	885	511	768	443	388	622
Masson pine	2607	1740	319	325	867	333	290
Chinese pine	1186	790	306	377	396	275	343
Yunnan pine	766	510	340	250	256	355	242
Other coniferous	1681	1125	471	323	556	414	327
Broadleaved							
Oak	4474	2980	706	875	1494	402	538
Birch	2201	1465	472	359	736	420	360
Poplar	3924	2615	396	367	1309	380	298
Robinia	830	550	188	248	280	193	245
Eucalypt	1036	690	261	296	346	195	248
Rubber-woods	701	465	298	241	236	370	296
Other hard-broad	3368	2245	488	549	1123	443	509
Other soft-broad	1729	1150	381	366	579	422	321
Mixed							
Coniferous	1898	1265	881	600	633	789	487
Conifer-broadleaved	4364	2910	650	606	1454	502	436
Broadleaved	13073	8715	732	870	4358	706	723

### Method

#### Model development

**Figure 1:**
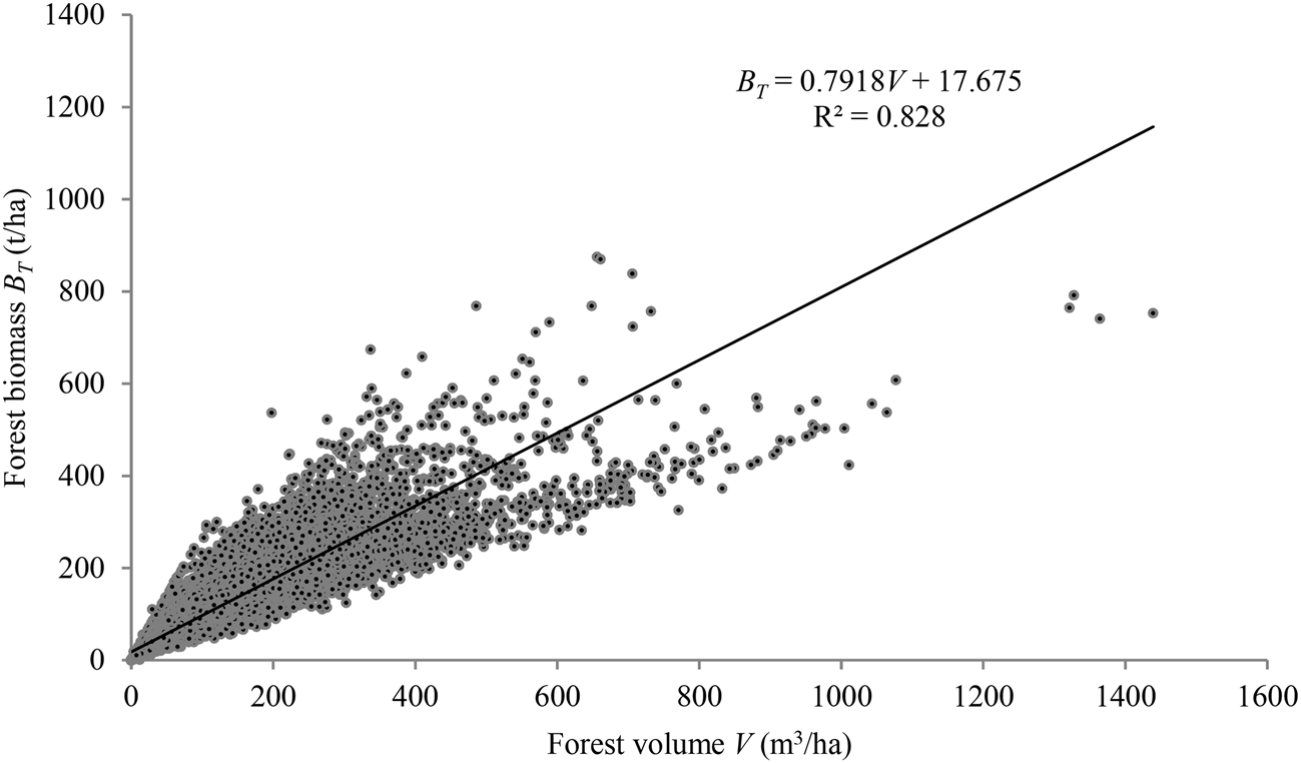
The scatterplot of forest biomass vs. forest volume for all 52,700 plots. The model was fitted from ordinary least square method.

Forest stand biomass is intrinsically tied to its volume, and this relationship has been extensively explored through volume-derived biomass models in prior studies^27,28,32,35,38,45–48^. Fang et al.^30^, in their research on 21 Chinese forest types, established a linear correlation between forest stand biomass and volume stock. This linearity is further corroborated by the scatterplot depicting the relationship between forest biomass and volume data per hectare across the 52,700 sample plots (as shown in Fig. 1).

While total biomass provides valuable insights, it is often essential to discern the distinction between above- and below-ground biomass. The latter's proportion to the former is termed the root-to-shoot ratio (RSR), which has been noted to exhibit variation across different forest types. Once the total forest biomass estimation is secured, the subsequent step involves calculating forest carbon storage. This is achieved by multiplying the biomass by the average carbon factor, which is typically either 0.5 or 0.47^2,29,30^. Yet, it's worth noting that distinct tree species and forest types might present varying carbon factors.

Given the recursive nature of the relationship between total biomass and either above-ground biomass or carbon storage, this study employed simultaneous equations with error-in-variables, a method previously harnessed for tree-level modeling^46–48^. The equations are articulated as:

1 $${{B}_{T}={a}_{0}+{b}_{0}V+\varepsilon }_{1},$$

2 $${{B}_{A}={c}_{0}{B}_{T}+\varepsilon }_{2},$$

3 $${C={d}_{0}{B}_{T}+\varepsilon }_{3}.$$

In these equations, *B*_*T*_ signifies total biomass per hectare (t/ha), *B*_*A*_ represents above-ground biomass (t/ha), and *C* denotes carbon storage (t/ha). *V* is the stand volume (m^3^/ha), and *a*_0_, *b*_0_, *c*_0_, *d*_0_ are the model’s parameters, *ε*_1_*, ε*_2_*, ε*_3_ are the error items, which are postulated to adhere to a normal distribution, averaging to zero.

By dividing Eq. (1) by *V*, a stand biomass conversion factor (BCF) model is derived:

4 $${BCF={B}_{T}/V={b}_{0}+{a}_{0}/V+\varepsilon }_{4}.$$

Within this context, BCF amalgamates three parameters: basic wood density (WD), biomass expansion factor (BEF) and the root-to-shoot ratio (RSR). This is consistent with the constructs proposed in the IPCC Guidelines for national greenhouse gas inventories^2^, wherein BCF = WD*BEF*(1 + RSR). The *d*_0_ parameter in Eq. (3) aligns with the carbon factor (CF). Additionally, drawing from the *c*_0_ parameter in Eq. (2), one can derive the RSR as:

5 $${RSR={B}_{B}/{B}_{A}={(1-c}_{0})/{c}_{0}+\varepsilon }_{5}.$$

Given the heteroscedastic nature of the data concerning forest biomass, carbon storage, and forest volume, the study recommends the adoption of the weighted regression method^45^. The weight function employed in this analysis was defined as *w* = 1/*V*^0.5^. Using the ordinary least square (OLS) method, without accounting for this heteroscedasticity, could inadvertently introduce biases. This is rooted in the premise that OLS methodological application is contingent upon homoscedasticity—one of its foundational assumptions. Additionally, in the light of the intertwined relationship between total and above-ground biomass or carbon storage, it becomes imperative to utilize simultaneous equations paired with error-in-variables for accurately fitting the models outlined in Eqs. (1) through (3)^45–48^.

#### Model evaluation

Six indices were used to evaluate the models: coefficient of determination (R^2^), standard error of the estimate (SEE), total relative error (TRE), average systematic error (ASE), mean prediction error (MPE), and mean percentage standard error (MPSE)^47–49^. TRE, ASE, MPE and MPSE are calculated as follows:

6 $$TRE = \sum {\left( {y_{i} - \hat{y}_{i} } \right)} /\sum {\hat{y}_{i} } \times 100$$

7 $$ASE = \sum {\left( {y_{i} - \hat{y}_{i} } \right)/\hat{y}_{i} } /n \times 100$$

8 $$MPE = t_{\alpha } \cdot \left( {SEE/\overline{y}} \right)/\sqrt n \times 100$$

9 $$MPSE = \sum {\left| {\left( {y_{i} - \hat{y}_{i} } \right)/\hat{y}_{i} } \right|} /n \times 100$$

In these equations, *y*_*i*_ are observed values, $\hat{y}_{i}$ are estimated values, $\overline{y}$ is the mean of observed values, *n* is the number of plots, and *t*_*a*_ is the *t*-value at confidence level *a*. For the developed models, the values of six indices above were calculated out and to be used for model evaluation.

### Results

For the purpose of this study, data from 35,120 plots spanning 20 distinct forest types were employed to fit models (1) through (3) via the application of simultaneous equations with error-in-variables. The resulting fit and its associated statistical assessment for model (1) are elucidated in Table 2. It is noteworthy that, when comparing the standard error of the estimate (SEE) for models (2) and (3) with model (1), significant discrepancies were observed. Such variations in the SEE provide insights into the consistency and reliability of the models. However, when examining the other five evaluative indices, only marginal differences were discerned. Owing to their minimal variation, these indices have been omitted from Table 2 for clarity and brevity.

**Table 2:** The parameter estimates and evaluation indices of biomass and carbon models for 20 forest types.

Forest types	Parameter estimates					Evaluation indices of model (1)					
	*a*_0_	*b*_0_	*c*_0_	*d*_0_(CF)	RSR	*R*^*2*^	*SEE*/m^3^	*TRE*/%	*ASE*/%	*MPE*/%	*MPSE*/%
Coniferous											
Fir	4.1095	0.5976	0.8181	0.4951	0.2223	0.871	47.62	− 0.04	2.82	2.22	17.37
Spruce	1.3879	0.7168	0.7991	0.4901	0.2514	0.913	29.97	− 0.02	5.79	1.24	15.77
Larch	0.6986	0.8262	0.7794	0.4888	0.2830	0.936	15.10	− 0.02	3.41	0.83	13.36
Chinese fir	0.5743	0.7120	0.8109	0.4969	0.2332	0.936	12.07	− 0.01	4.43	0.87	14.06
Cypress	0.4545	1.5341	0.8007	0.5013	0.2489	0.947	22.57	0.00	3.02	1.65	16.48
Masson pine	1.4282	0.9768	0.8297	0.5162	0.2053	0.953	11.28	0.00	1.31	0.67	9.95
Chinese pine	0.2112	1.1235	0.8098	0.5130	0.2349	0.966	16.17	0.00	− 0.63	1.66	15.72
Yunnan pine	0.9158	0.6501	0.8453	0.5047	0.1830	0.973	11.89	0.00	3.98	1.71	13.81
Other coniferous	1.0721	0.8303	0.8041	0.5009	0.2436	0.946	20.86	− 0.01	6.35	1.64	20.35
Broadleaved											
Oak	0.7196	1.2948	0.7930	0.4810	0.2610	0.917	26.74	− 0.01	2.95	0.85	17.44
Birch	0.7507	1.0118	0.7821	0.4867	0.2786	0.898	17.34	0.01	1.28	1.02	14.48
Poplar	0.2945	0.8978	0.8246	0.4725	0.2127	0.934	12.18	0.00	0.23	0.78	13.19
Robinia	0.3864	1.5499	0.7833	0.4838	0.2767	0.933	10.83	− 0.02	3.33	2.02	14.83
Eucalypt	0.3330	1.1740	0.7793	0.5238	0.2832	0.962	8.98	0.00	1.32	1.18	8.88
Rubber-woods	0.2401	0.8679	0.7981	0.4956	0.2530	0.989	4.36	− 0.01	3.74	0.57	6.83
Other hard-broad	0.3454	1.2130	0.7880	0.4856	0.2690	0.933	14.37	− 0.01	2.94	1.17	17.67
Other soft-broad	0.6534	0.9920	0.7954	0.4873	0.2572	0.897	18.63	− 0.02	4.83	1.79	19.65
Mixed											
Coniferous	3.3612	0.8348	0.8088	0.5014	0.2364	0.919	19.98	− 0.01	2.41	1.25	14.16
Conifer-broadleaved	2.2575	0.9560	0.7961	0.4920	0.2561	0.892	20.73	− 0.04	3.84	0.90	15.85
Broadleaved	1.3944	1.1086	0.7938	0.4835	0.2598	0.916	22.69	− 0.03	3.36	0.47	15.48

All parameter estimates are significant at the level *a* = 0.01. Parameter *d*_0_ is equivalent to the average carbon factor (CF) of each forest type, and RSR is the average root-to-shoot ratio resulting from Eq. (5).

In a comprehensive examination of the evaluation metrics presented in Table 2, several key insights about the model's performance emerged. The coefficient of determination R^2^ consistently recorded values above 0.87, illustrating the model's high level of explanatory power. The total relative error (TRE) was observed to be close to zero, indicating minimal discrepancies between the observed and predicted values across the models. Moreover, the average systematic error (ASE) was predominantly confined within a range of ± 7%. Interestingly, for 18 of the 20 forest types under study, the ASE remained even more constrained, falling within ± 5%. Additionally, the mean prediction error (MPE) across all types was kept below 3%, and for eight forest types, it was remarkably less than 1%. On evaluating the mean percentage standard error (MPSE), it was found that values for most of the forest types fell within a bracket of 10% to 20%. It's worth noting that only one forest type exhibited an MPSE exceeding 20%, while three types demonstrated values under the 10% mark.

Further, in a subsequent phase of analysis, data from 17,580 plots, as delineated as validation samples in Table 1, were subjected to an independent validation. The results, particularly the TRE and ASE values, are detailed in Table 3. A key observation from this validation was that, for model (3), the TRE exceeded ± 3% exclusively for the 'other coniferous' category. In all other instances, the ASE for each forest type remained tightly bound within a range of ± 5%.

**Table 3:** The independent validation results of biomass and carbon models for 20 forest types.

Forest type	TRE/%			ASE/%		
	Model (1)	Model (2)	Model (3)	Model (1)	Model (2)	Model (3)
Coniferous						
Fir	− 0.09	0.02	− 0.11	− 0.03	0.40	− 0.02
Spruce	− 1.56	− 1.40	− 1.57	− 3.31	− 3.38	− 3.32
Larch	0.29	0.24	0.27	− 3.13	− 2.06	− 3.14
Chinese fir	0.29	0.32	0.38	0.45	0.46	0.52
Cypress	0.43	0.42	0.38	2.05	2.20	2.18
Masson pine	− 0.44	− 0.56	− 0.46	3.61	2.95	3.56
Chinese pine	0.60	0.73	0.71	3.92	3.34	3.92
Yunnan pine	1.10	1.01	1.19	− 0.93	− 1.11	− 1.04
Other coniferous	− 2.93	− 2.90	− 3.16	2.03	2.36	2.16
Broadleaved						
Oak	− 0.61	− 0.46	− 0.57	1.60	3.06	1.50
Birch	0.44	0.47	0.43	2.91	4.08	2.91
Poplar	− 0.72	− 0.75	− 0.75	3.32	4.31	3.38
Robinia	2.16	2.09	2.11	3.48	4.47	3.41
Eucalypt	1.19	1.19	1.22	0.97	1.72	1.15
Rubber-woods	− 0.05	− 0.08	− 0.02	− 3.32	− 2.72	− 3.29
Other hard-broad	− 0.87	− 0.92	− 0.97	1.52	2.64	1.03
Other soft-broad	0.57	0.78	0.48	3.57	4.87	3.45
Mixed						
Coniferous	0.66	0.48	0.70	1.58	1.33	1.51
Conifer-broadleaved	− 0.16	− 0.30	− 0.21	1.58	1.73	1.45
Broadleaved	− 0.10	− 0.09	− 0.08	0.78	1.70	0.63

Considering the aforementioned metrics and observations, it is evident that the stand biomass and carbon storage models for the 20 forest types display an admirable performance. Such precision and reliability underscore their potential in providing an accurate and robust framework for estimating forest biomass and carbon storage at the stand level.

## Estimating changes of forest carbon storage

### Data collection

The dataset for this study is primarily sourced from the records of the nine National Forest Inventories (NFI) as well as forest area and volume data documented prior to the first NFI^50–55^. This compilation encompasses provincial data for dominant tree species or forest types, along with specific datasets for bamboos and sparse forest.

### Estimation methodology

#### Estimation by classification

The time span covering the 1st to the 9th NFI witnessed two significant alterations in the definition of a forest. Initially, before the 5th NFI was embarked upon, there was a revision in the canopy closure parameter, shifting it from above 0.3 (excluding 0.3, akin to more than 35%) to above 0.20. A subsequent modification, just before the 6th NFI concluded, entailed the inclusion of specifically defined shrubs in the forest area and forest coverage measurements^56^.

In striving for a data alignment compatible with global norms, the study embraced the FAO’s forest definition^57,58^. Accordingly, only arboreal forests, bamboos, and rubber-woods were included, while the specially defined shrubs were left out. The adopted canopy closure standard for forests stood at more than 10%. Sparse forests from prior NFIs were incorporated, defined by canopy closures ranging between 10 and 35% (1st to 4th NFI) and 10–19% (5th to 9th NFI).

The methodology further delves into specifics:

1. Arboreal forest carbon storage. Leveraging the earlier developed models for 20 forest types, estimations for biomass and carbon storage were derived using the area and volume metrics for varying forest types.
2. Bamboos’ carbon storage. The 9th NFI provided a foundation using the individual bamboo plant biomass model and the 0.5 carbon factor. Proportional methods, relying on bamboo area from previous NFIs, were employed to compute the biomass and carbon storage.
3. Sparse forest carbon storage. Historical data showcasing the volume proportion of sparse to arboreal forests informed the proportional method used for determining their biomass and carbon storage.

#### Treatment of incomparable data

Ensuring that forest carbon storage transitions across distinct periods were accurately portrayed necessitated some recalibrations. Built on the data from earlier NFIs, these adjustments were steered by a thorough evaluation of data comparability and cohesion.

1. Tibet’s forest data. Till the 6th NFI, Tibet had been surveyed for its forests only twice (in 1977 and 1991). A stark contrast was observed in its forest area and volume, which lagged behind the 2001 records. Given an inferred mild reduction in forest resources pre-2001, data from the 1st to the 5th NFI, along with 1949 and 1962 datasets, underwent necessary adjustments.
2. Taiwan’s forest data. Four forest inventories had been executed in Taiwan across 1957, 1976, 1992, and 2012. While aggregating national statistics, the 1976 dataset represented the 2nd, 3rd, and 4th NFIs. Subsequently, the 1992 data echoed the findings of the 5th through the 8th NFIs. The 1957 and 2012 records, on the other hand, paralleled the 1st and 9th NFIs respectively. Ensuring an objective reflection of forest carbon storage transitions, interpolative and extrapolative techniques were applied to the NFI datasets, grounding them on the quartet of Taiwan's inventories.
3. Other data amendments. The second NFI marked the adoption of the Continuous Forest Inventory (CFI) methodology, rendering the 1st NFI data, as well as the 1949 and 1962 records, somewhat incongruent. Thus, employing a mix of the 2nd and 3rd NFIs' dynamic datasets and the trend analysis for 1949 and 1962^50^, certain figures from the 1st NFI and the two mentioned years underwent revisions and augmentations.

### Results

Using the aforementioned estimation methods for biomass and carbon storage, combined with the processing of incomparable data, we charted the evolution of forest metrics in China over a span of 70 years from 1949 to 2018. Concurrently, we also traced the changes in metrics specific to planted forests from the 2nd to the 9th NFI, covering an approximate timeline of 40 years from 1977 to 2018, as detailed in Table 4.

**Table 4:** The estimation results of forest biomass and carbon storage in different periods in China.

Period	FA (10^6^ ha)	FV (10^9^ m^3^)	FB (Pg)	FC (Pg)	FD (Mg/ha)	PA (10^6^ ha)	PV (10^8^ m^3^)	PB (Pg)	PC (Pg)	PD (Mg/ha)
1949	141.06	12.90	11.93	5.89	41.73	/	/	/	/	/
1950–1962	133.37	11.79	10.91	5.38	40.35	/	/	/	/	/
1973–1976	127.63	10.83	9.98	4.91	38.43	/	/	/	/	/
1977–1981	123.76	10.58	10.00	4.90	39.62	13.67	2.86	0.31	0.15	11.25
1984–1988	133.27	10.72	10.22	5.01	37.57	22.99	5.84	0.59	0.29	12.78
1989–1993	137.67	11.10	10.77	5.28	38.35	25.94	7.76	0.77	0.38	14.79
1994–1998	147.85	11.69	11.35	5.57	37.64	32.74	10.67	1.02	0.51	15.46
1999–2003	156.67	12.70	12.39	6.08	38.81	36.07	15.66	1.49	0.74	20.50
2004–2008	169.02	14.00	13.73	6.73	39.83	44.48	20.31	1.94	0.96	21.64
2009–2013	178.14	15.46	15.34	7.52	42.23	51.92	25.70	2.52	1.25	24.11
2014–2018	191.97	17.70	17.74	8.69	45.29	60.64	34.05	3.42	1.69	27.82

FA, FV, FB, FC, and FD are forest area, forest volume, forest biomass, forest carbon, and forest carbon density, respectively; and PA, PV, PB, PC, and PD are planted forest area, planted forest volume, planted forest biomass, planted forest carbon, and planted forest carbon density, respectively. Forest is the land with a tree canopy closure of more than 10%, including bamboos and rubber-woods, excluding specially defined shrubs.

From the insights of Table 4, it becomes evident that China's forest resources underwent a period of decline from the early years following the establishment of the People's Republic of China until the concluding years of the 1970s. The carbon storage metric mirrors this trend, beginning at 5.89Pg and decreasing to its lowest recorded level at 4.90Pg due to extensive forest cutting for the rapid economic and social development. Being a developing nation at the time, China's economic progression necessitated large volumes of wood^50^. Venturing into the era post-reform, we witnessed a resurgence in plantation growth. Coinciding with the large-scale afforestation initiatives that swept across the nation, the carbon storage values associated with these plantations surged from a meager 0.15Pg in the late 1970s to an impressive 1.69Pg by the time of the 9th NFI. An additional significant observation centered around the end of the twentieth century. As China rolled out pivotal forestry programs, such as the natural forest protection and the transition of farmlands back to their original forested state, there was a marked acceleration in the growth of forest resources. This uptick is quantified with the carbon storage values rising from its previous low of 4.90Pg in the late 1970s to the highest of 8.69Pg recorded in the 9th NFI.

**Figure 2:**
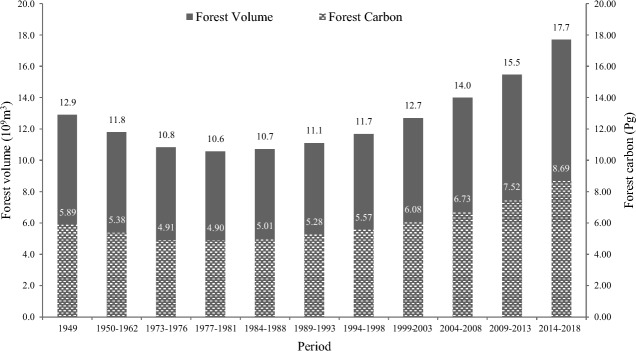
The change trend of forest volume and carbon storage in China.

Stretching our lens over the entire 70-year span, we discern a distinctive U-shaped trajectory in both forest volume and carbon storage, which is further exemplified in Fig. 2. Narrowing our scope to just the last four decades, the increment in forest carbon storage is quantified at 3.79Pg. Of this, natural forests contributed 2.25Pg, and planted forests added 1.54Pg, translating to respective proportions of 59% and 41%. Both afforestation and natural forest protection have contributed greatly to the growth of forest carbon storage in China.

## Discussions

### Biomass models

In our introduction, we highlighted the limited number of biomass models for forest types—specifically larch, Chinese fir, and Chinese pine—established by Fang et al.^30^ that had modeling samples exceeding 30. To offer a comparative analysis, we assessed the biomass models crafted by Fang et al.^30^, Wang et al.^32^ and Zhang et al.^41^, utilizing the data from all sample plots of these three forest types, as depicted in Table 5.

**Table 5:** The comparison of estimation results of different biomass models for three forest types.

Forest types	TRE (%)	ASE (%)	Biomass models	Source
Larch	11.62	168.64	*B* = 33.8060 + 0.6096*V* (*n* = 34, *R*^2^ = 0.82)	Fang et al.^30^
	− 11.58	− 8.92	*B* = *V*/(1.1111 + 0.0016*V*) (*n* = 39, *R*^2^ = 0.916)	Wang et al.^32^
	− 8.16	− 3.84	*B* = 1.4091*V*^0.8752^ (*n* = 241, *R*^2^ = 0.970)	Zhang et al.^41^
Chinese fir	− 6.26	215.86	*B* = 22.5410 + 0.3999*V* (*n* = 56, *R*^2^ = 0.95)	Fang et al.^30^
	− 12.58	− 10.01	*B* = *V*/(1.2917 + 0.0022*V*) (*n* = 70, *R*^2^ = 0.910)	Wang et al.^32^
	− 14.82	− 7.14	*B* = 1.2877*V* ^0.8427^ (*n* = 88, *R*^2^ = 0.929)	Zhang et al.^41^
Chinese pine	− 25.31	23.14	*B* = 5.0928 + 0.7554*V* (*n* = 82, *R*^2^ = 0.96)	Fang et al.^30^
	− 28.33	− 22.18	*B* = *V*/(1.0529 + 0.0020*V*) (*n* = 147, *R*^2^ = 0.937)	Wang et al.^32^
	− 19.99	− 6.70	*B* = 1.7969*V*^0.8416^ (*n* = 699, *R*^2^ = 0.929)	Zhang et al.^41^

**Figure 3:**
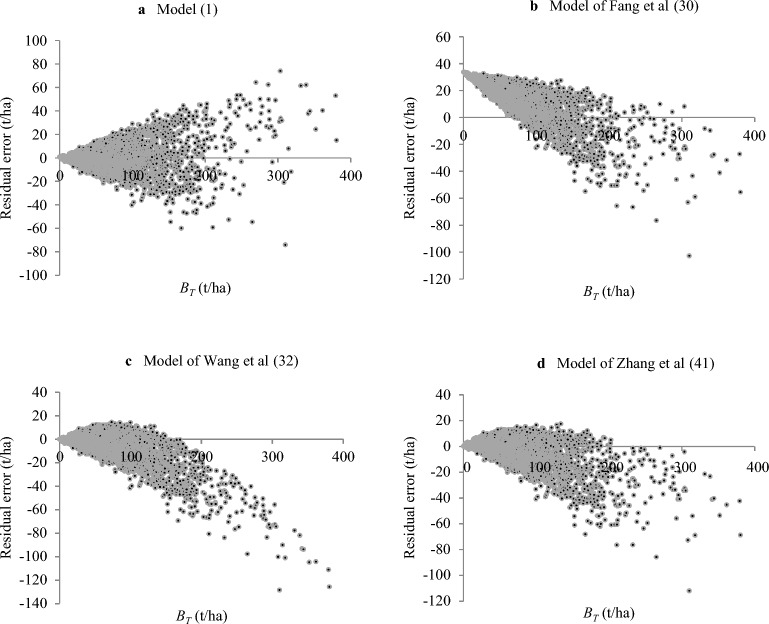
Comparison of residual error plots between model (1) and other three models for larch. (**a**) Model (1); (**b**) Model of Fang et al.^30^; (**c**) Model of Wang et al.^32^; (**d**) Model of Zhang et al.^41^.

A close examination of the evaluation indices TRE and ASE from Table 5 reveals a noteworthy trend: as sample size increases, the precision of the three model sets generally improves. Broadly speaking, Zhang et al.’s models^41^ show a superior performance over those by Wang et al.^32^, and Fang et al.’s models^30^ exhibit the most substantial errors. Dissecting the parameters from the models formed in our study highlights that Fang et al.'s models possess larger intercept parameters but smaller slope parameters, leading to considerably abnormal ASE values as illustrated in Fig. 3.

Several factors can potentially explain these disparities. Foremost, a biased estimation method could be a contributing factor. Relying on ordinary regression instead of weighted regression, especially when faced with heteroscedasticity, may result in such skewed outcomes. Additionally, both the magnitude and the structural quality of the sample cannot be dismissed as influencing variables. Conventionally, for statistical hypotheses to be valid, the sample size should exceed 50. As underscored by the key index in Eq. (9)^49^, an uptick in sample size is inversely related to MPE. The quality of sample structure is another crucial determinant, further elucidated by certain studies^42,47^. Other elements such as tree species, age, and regional variability can also affect model performance. For instance, when we divide larch forest plots into three regional subsets, outcomes from regional models differ from the national model.

Notably, the challenge of uncertainty in forest biomass and carbon stock estimations is not an isolated phenomenon in China but is witnessed globally. The essence of modeling underscores the significance of garnering ample samples, employing the correct parameter estimation method, and employing a diverse range of evaluation indices.

Figure 3 sheds light on the residual errors in the biomass model (1) alongside the other three biomass models for larch. Distinctively, owing to the intercept parameter a_0_ = 33.806 in Fang et al.’s model^30^, a larch forest stand with a volume of 0 m^3^/ha exhibits a biomass of an elevated 33.806 t/ha. This inevitably leads to biases in biomass estimates for larch forest stands. It's evident that not only is the total biomass consistently underestimated, but the same issue of biases persists. This pattern mirrors the residual errors found in biomass models for other forest types like Chinese fir and Chinese pine. However, to optimize space, these patterns are not presented here.

### Carbon changes

To further elucidate the disparities between the outcomes of previous studies and our own, we examined the fluctuations in China's forest carbon storage across various time frames as estimated by Fang et al.^30^, Zhou et al.^38^ and Zhang et al.^40^. A comparison was then made with the data found in Table 4 of our study (refer to Table 6).

**Table 6:** The comparison of estimation results of forest carbon storage changes from different sources.

Period	Fang et al.^30^	Zhou et al.^38^	Zhang et al.^40^	This study
1949	5.06	/	4.38	5.89
1950–1962	4.58	/	3.98	5.38
1973–1976	4.44	3.0	3.79	4.91
1977–1981	4.38	3.2	3.69	4.90
1984–1988	4.45	3.3	3.71	5.01
1989–1993	4.63	3.6	4.08	5.28
1994–1998	4.75	4.1	4.56	5.57
1999–2003	/	4.9	5.43	6.08
2004–2008	/	5.3	6.10	6.73
2009–2013	/	5.9	6.81	7.52
2014–2018	/	/	7.97	8.69

Given that all four investigations derive from the NFI data, the variations in results are relatively minimal. Specifically, the alterations in forest carbon storage in China across different periods as discerned by Zhang et al.^40^ largely mirror our findings, although their values are systematically lower. Besides the models' inherent negative bias, several distinctions can be noted. First, Zhang et al.'s forest data for 1949 and 1950–1962 omitted Tibet. Second, data for 1977–1981, 1984–1988, 1989–1993, and 1994–1998 only partially included Tibet. Third, their data across all periods excluded both bamboos and sparse forest.

Likewise, Zhou et al.'s^38^ forest carbon storage figures across eight NFIs in China consistently undershoot, and the lowest value manifests not in 1977–1981 but in 1973–1976—a discrepancy in the observed trend. Beyond employing diverse methodologies, this difference can also be traced back to certain contrasts with our study. Zhou et al. restricted their scope to the mainland, excluding Taiwan; only partially included Tibet in the data from the 1st to the 5th NFI; and omitted bamboos and sparse forest from each period's data.

The trend in forest carbon storages from 1949 to 1998 as determined by Fang et al.^30^ aligns perfectly with our study, yet their values are also systematically reduced. The authors neither supplied intermediate data nor delineated what was included or excluded. Beyond utilizing disparate models, the causes for this underestimation may include the following: an exclusion of sparse forest in each period's data; a possible omission of bamboos; and a likely exclusion of both Taiwan and parts of Tibet.

In summary, the aforementioned three studies appear to have overlooked the ramifications of changes in forest definitions and disparities in statistical scope across different periods while employing NFI data. This oversight led to a systematic underestimation of forest carbon storage across various time frames, with particularly pronounced underestimations in the earlier periods.

## Conclusions

Utilizing the data acquired from 52,700 permanent plots during the 9th NFI in China, we established biomass and carbon storage models for 20 distinct forest types. This was achieved through the application of simultaneous equations with error-in-variables. Furthermore, leveraging data pertaining to forest area and volume across different timeframes, we delineated the shifts in forest carbon storage over a 70-year span and charted the alterations in planted forest carbon storage over the past 40 years. From these results, several significant conclusions emerge:

1. The biomass models for 20 forest types, derived from volume data in our study, demonstrated robust predictive capability. With an R^2^ exceeding 0.87 and a MPE under 3%, these models offer a foundational basis for accurate estimation of the status and changes in forest carbon storage, both nationally and regionally.
2. We assessed the validity of three existing sets of biomass models using our dataset. Although all models exhibited notable biases, the accuracy of their predictions appeared to enhance as the modeling sample size grew.
3. Historical data reveals that China's forest carbon storage stood at 5.89Pg in 1949, dipped to a nadir of 4.90Pg by the late 1970s, and subsequently rose to 8.69Pg by the time of the 9th NFI. Over the past seven decades, the trajectory of China's forest carbon storage can be best described as U-shaped.
4. The last 40 years have witnessed a surge of 3.79Pg in China's forest carbon storage. This growth encompasses an increment of 2.25Pg in natural forests and 1.54Pg in planted forests. This trend underscores the pivotal roles of both afforestation initiatives and the protection of natural forests in bolstering China's forest carbon storage.

## Data Availability

Data will be made available upon request to W.S. Zeng.
